# Living off the fat of the plant: Powdery mildew fungi thrive on host thylakoid lipids

**DOI:** 10.1093/plcell/koaf044

**Published:** 2025-04-07

**Authors:** Jan Wilhelm Hübbers

**Affiliations:** Assistant Features Editor, The Plant Cell, American Society of Plant Biologists; Unit of Plant Molecular Cell Biology, Institute for Biology I, RWTH Aachen University, 52056 Aachen, Germany

“Eat healthy fats!” is a familiar mantra to promote a balanced diet. It highlights the human need for polyunsaturated fatty acids such as omega-3. Biochemically, omega-3 describes a double bond within a fatty acid chain 3 atoms away from the terminal methyl group. A common example of an omega-3 fatty acid is α-linolenic acid, a carboxylic acid with an 18-carbon chain and 3 cis double bonds (18:3). Essential fats such as α-linolenic acid are vital for human metabolism, and we typically obtain these lipids from plant-based foods like nuts, seeds, and oils.

Interestingly, in this respect, humans have something in common with powdery mildew fungi. These notorious plant parasites are named after the eponymous symptoms they cause on the surface of above-ground plant parts. As obligate biotrophs, powdery mildew fungi rely on living host cells for nutrients, which they extract using specialized feeding structures called haustoria. These structures form 1 day after infection and resemble tiny, inflated laboratory gloves within host epidermal cells. By 3 to 7 days post infection, the fungi produce spores on the plant surface that create the characteristic powdery appearance (Kuhn et al. 2016). The spores are filled with plant-derived lipid bodies that provide energy reservoirs for future infections (Jiang et al. 2017).

In new work, **Hang Xue, Johan Jaenisch, and colleagues (Xue et al. 2025)** unmasked how powdery mildew fungi manipulate host plant metabolism to extract thylakoid lipids for their own benefit. Using the *Arabidopsis thaliana*-*Golovinomyces orontii* pathosystem, the authors investigated the accumulation of lipids in infected rosette leaves. They observed a 3.5-fold increase in triacylglycerols (TAGs) in infected wild-type *A. thaliana* plants 12 days post inoculation, with a notable enrichment of unsaturated fatty acids, including 18:2 and 18:3. The authors then searched for 16:3 fatty acids, which are specific to the chloroplast. TAGs containing these fatty acids were indeed enriched in infected leaves, suggesting chloroplasts as the primary lipid source for powdery mildew fungi.

TAGs are storage lipids composed of 3 fatty acids attached to a central glycerol backbone. In eukaryotes, the final and rate-limiting step of TAG synthesis is catalyzed by diacylglycerol transferases (DGAT) in the endoplasmic reticulum (Xu et al. 2020). The authors tested whether loss-of-function of the canonical *A. thaliana* DGATs, DGAT1 and DGAT2, hinders powdery mildew spore production. Strikingly, after infection with *G. orontii*, *dgat2* mutants showed no significant difference in spore accumulation, while *dgat1* mutants showed enhanced powdery mildew spore development compared with Col-0 wild-type plants (see Figure).

**Figure.**
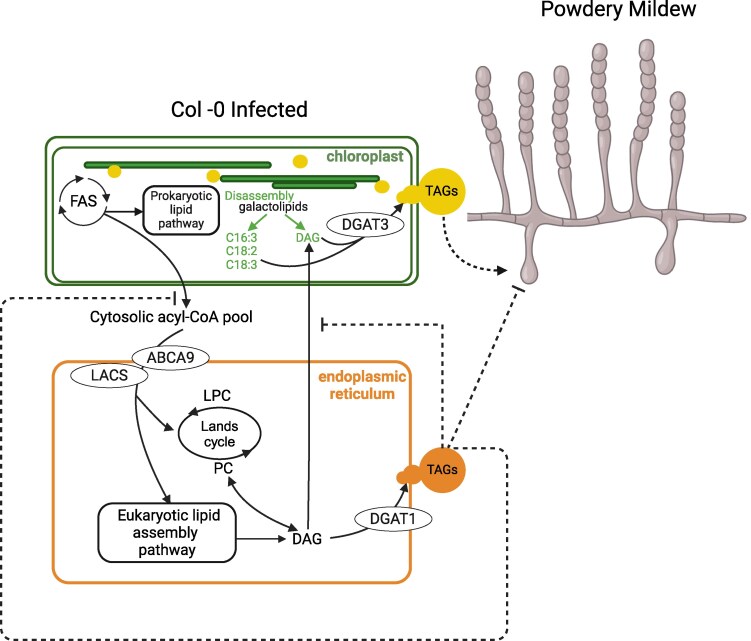
Powdery mildew fungi modulate the host lipid metabolism during their asexual reproduction on Arabidopsis. DGATs catalyze the final and rate-limiting step during TAG metabolism. During powdery mildew infection, the *A. thaliana* chloroplast-resident DGAT3 enzyme uses thylakoid lipids to make TAGs for fungal spore production. The canonical DGAT1 enzyme acts in an antagonistic pathway in the endoplasmic reticulum. Reprinted from Xue et al. (2025), Figure 7.

Unlike DGAT1 and DGAT2, *A. thaliana* DGAT3 has an N-terminal chloroplast transport signal. The authors confirmed chloroplast localization using fluorophore-labeled DGAT3 in *Nicotiana benthamiana* and found that DGAT3 knockdown or knockout reduced powdery mildew spore production by 20% to 50%. Notably, DGAT3 prefers 18:2 and 18:3 substrates (Hernández et al. 2012), which were considerably reduced in chloroplast TAGs of infected *dgat3* mutants compared with infected wild-type leaves.

Plants deprived of DGAT3 did not show constitutive activation of plant defense. Thus, the authors concluded that reduced spore production on *dgat3* mutants is due to decreased TAG production in chloroplasts. By contrast, DGAT1 reduces spore production by limiting substrates for chloroplast TAG synthesis (see Figure) and/or by contributing to the presence of endoplasmic reticulum-derived lipid droplets, containing defense compounds.

In this work, the authors uncovered the role of DGAT3 in obligate biotrophy and lipid metabolism. Among other findings, their results open the interesting perspective of leveraging powdery mildew infection to study plant lipid metabolism. This approach may also support engineering plant TAG profiles enriched in fatty acids essential for a balanced human diet.

## Recent related articles in *The Plant Cell*

- Dong et al. (2025) used single-cell mass spectrometry and gene editing to produce an automated pipeline to engineer and profile increased lipid production in plant cells and whole plants.
- Huebbers et al. (2024) uncovered isoform-specific interactions between MILDEW RESISTANCE LOCUS O (MLO) proteins and exocyst component EXO70 proteins, affecting both modulation of trichome cell wall biogenesis and powdery mildew susceptibility.
- Dodds et al. (2024) reviewed the main principles of plant immunity, emphasizing key scientific milestones.
- Ostermeier et al. (2024) reviewed thylakoid membrane architectures in phototrophs, aiming to define the underlying principles guiding the evolution of these bioenergetic membranes.
